# Telehealth Use Among Medicaid-Enrolled Children with Sickle Cell Disease Before and During the COVID-19 Pandemic

**DOI:** 10.3390/healthcare13131519

**Published:** 2025-06-25

**Authors:** Gloria N. Odonkor, Hyeun Ah Kang, Ange Lu, Robert C. Mignacca, Alicia Chang, Kenneth A. Lawson

**Affiliations:** 1 College of Pharmacy, The University of Texas at Austin, Austin, TX 78712, USA; 2 Children’s Blood and Cancer Center at Dell Children’s Hospital, Austin, TX 78723, USA; 3 The Department of Pediatrics, Dell Medical School, The University of Texas at Austin, Austin, TX 78723, USA

**Keywords:** telehealth, sickle cell disease, COVID-19, Medicaid, children

## Abstract

**Background/Objectives:** Children with sickle cell disease (SCD) often experience limited access to care, contributing to poor health outcomes. Patient-level predictors and outcomes associated with telehealth use among Medicaid-enrolled children with SCD remain unknown. This study aims to (1) analyze telehealth trends before and during the pandemic (March 2020–March 2022), (2) identify patient-level predictors of telehealth use, (3) assess its association with care continuity and health outcomes, and (4) identify physician specialties involved in telehealth visits. **Methods:** Using Texas Medicaid claims (March 2017–March 2022), we conducted a retrospective analysis of children aged 1–18 with ≥3 SCD-related claims. Monthly trends in outpatient visits (in-person and telehealth) were visualized from March 2019 to March 2022. Multivariable regression models examined predictors of telehealth use and associations with ≥10 hydroxyurea fills, emergency department (ED) visits, and hospitalizations, adjusting for age, sex, regions with SCD clinics, and prior healthcare utilization. **Results**: Among 903 included patients (mean [SD] age = 10.4 [4.1], 52.6% male), 59.4% had ≥1 telehealth visits between March 2019 and March 2022. Telehealth use peaked between March 2020 and May 2020, then gradually declined. Children with ≥10 SCD-related outpatient visits 1 year before the lockdown (March 2019–February 2020) had 77.4% higher odds of using telehealth compared to those with 0–4 visits (OR = 1.774, 95% CI = 1.281–2.457, *p* = 0.0006), while controlling for sociodemographic characteristics. However, SCD-related telehealth use during the pandemic was not associated with either ≥10 hydroxyurea fills or reduced ED visits. Prior healthcare utilization remained a strong predictor of both outcomes. The majority of telehealth visits were conducted at multispecialty clinics (74%). **Conclusions:** Telehealth use surged early in the pandemic but later declined among Texas Medicaid-enrolled children with SCD. Children with high healthcare needs adopted telehealth, but this did not impact care continuity or extensive healthcare utilization. While maintaining telehealth access, other measures should be implemented to improve access and outcomes for this vulnerable population.

## 1. Introduction

Sickle cell disease (SCD) is a genetic disorder causing red blood cells to harden and form a crescent “sickle” shape, leading to complications such as stroke, chronic pain, infections, and kidney disease [1,2]. Treatment for children with SCD includes preventive therapies (antibiotics, immunizations, and transcranial screenings), disease-modifying therapies (hydroxyurea, L-glutamine, and crizanlizumab), pain medications, blood transfusions, and emerging gene therapies [3]. Despite these available therapies, patients with SCD have a life expectancy of 52.6 years, with approximately 100,000 people being affected in the United States [4], over 90% of whom are non-Hispanic Black or African American [5]. Although patients with SCD have extensive healthcare needs, limited access to specialized care, particularly in low-income or rural areas [6,7], contributes to poor outcomes and higher mortality [7], which is exacerbated by racial inequities and discrimination, especially in pain management [8].

With the emergence of the COVID-19 pandemic in 2019, there was an increase in the use of telehealth [9], which employs telecommunication technologies to support remote healthcare services by healthcare professionals [10]. As the use of telehealth expands, there is a potential opportunity to improve healthcare access, particularly for low-income, minority, rural communities, and for patients with SCD [9,11]. In general, telehealth visits are best suited for routine follow-ups, medication management, and psychosocial support, while in-person visits are often required for physical examinations, lab monitoring, and acute symptom evaluation [12,13]. The appropriate balance between telehealth and in-person care can vary according to disease, and in the context of children with SCD—where patients often require comprehensive disease and developmental monitoring and management—both the clinical needs and preferences of patients/caregivers and providers may also influence the mode of care delivery [14]. According to a study using Michigan Medicaid claims data, telehealth use increased during 2020 among children and adolescents with SCD, with 49% of visits involving hematologists [15], suggesting that telehealth may be a potential avenue for improving access to both general and specialized care. Another study analyzing claims data from four state Medicaid programs confirmed increased telehealth use among patients with SCD in 2020 and showed greater acute care utilization (outpatient, emergency department [ED], and inpatient) among telehealth users compared to non-users [16].

However, there is limited information on patient-level factors linked to telehealth use, such as age, sex, urban vs. rural residence, proximity to SCD clinics, and baseline healthcare utilization for SCD, especially when using adjusted analysis. Children with SCD living in areas with limited access to SCD clinics may benefit from telehealth. However, so far, it is unclear if these patients actually used these services. Additionally, the cross-sectional study design and limited follow-up during the early pandemic (March 2020–December 2020) in the study by Reeves et al. limited the analysis to acute care utilization during the same timeframe as telehealth use, without accounting for other potential confounders [16]. Moreover, little is known about the association of telehealth use with care continuum, which is essential for managing SCD.

The goal of this study is to address these gaps by analyzing Texas Medicaid data, which includes the third-largest SCD population in the United States [17]. Specifically, this study aims to (1) examine telehealth visit trends among children with SCD before and during the extended pandemic period (March 2020–March 2022), (2) identify patient-level factors associated with all-cause telehealth use, (3) assess the association between SCD-related telehealth use and care continuum (≥10 hydroxyurea fills) and extensive care (ED visits and hospitalizations) utilization, and (4) identify physician specialties associated with telehealth visits.

## 2. Materials and Methods

### 2.1. Study Design and Data Source

This retrospective cohort study used the Texas Medicaid claims data of pediatric patients with SCD between April 2017 and March 2022. The data included de-identified unique patient numbers, demographic and eligibility information, outpatient and inpatient medical claims, and pharmacy claims. Due to incomplete data, data from June 2020 was not used in the study. This study was deemed non-human subjects’ research by the Institutional Review Board at The University of Texas at Austin.

### 2.2. Patient Population

Texas Medicaid beneficiaries were included in the study if they (1) had at least 3 hospitalizations or outpatient visits associated with a diagnosis of SCD (International Classification of Diseases, Tenth Revision, Clinical Modification [ICD-10-CM] codes: D57, D57.x and D57.xx excluding D57.3 [sickle cell trait]) between April 2017 and March 2022, according to the CMS recommendation [18]; (2) were aged between 1 and 18 years during the study period (March 2019–March 2022); and (3) were continuously enrolled in Texas Medicaid during the study period. Subjects were excluded if they were diagnosed with cancer at any point between April 2017 and March 2022, or if they were eligible for both Medicaid and Medicare during the study period (Supplementary Table S1). For the objective of assessing the association between SCD-related telehealth use and healthcare utilization, individuals were included if they had at least one outpatient visit, either via telehealth or in-person, during the first year of the pandemic (March 2020–March 2021, excluding June 2020).

### 2.3. Study Variables

Differences in patient characteristics between telehealth users and non-users were examined. Telehealth users were identified using procedure codes and modifiers defined by the Texas Health and Human Services Commission [19] (Supplementary Table S2). All-cause monthly outpatient visit trends (in-person and telehealth) from March 2019 to March 2022 were graphically depicted. Demographic (age, sex, race/ethnicity, residential area) and clinical (pre-pandemic SCD-related outpatient visits from March 2019 to February 2020) characteristics were examined as potential factors associated with telehealth use, regardless of SCD diagnosis. Residential areas included regions with at least one healthcare facility with an SCD specialist listed by the Texas Department of State Health Services as a Pediatric Hematology Consultant (as of November 2023) [20] in the five largest cities in Texas (i.e., Houston, Fort Worth, Dallas, Austin, and San Antonio). Because 94.0% of the study population overlapped between the two aforementioned region categories, only the ‘Regions with an SCD clinic’ variable was used in multivariable models. The care continuum was defined as having 10 or more hydroxyurea claims in the first year of the pandemic (March 2020–March 2021, excluding June 2020). This definition was adopted because hydroxyurea is recommended by the 2014 National Heart, Lung, and Blood Institute (NHLBI) guidelines for reducing SCD-related complications, including vaso-occlusive pain episodes [21,22], and is expected to be taken continuously [20]. Extensive healthcare utilization included SCD-related ED visits and hospitalizations within 12 months of the first SCD-related telehealth visit (for telehealth users) or the first SCD-related in-person outpatient visit (for non-users). SCD-related ED visits and hospitalizations 12 months before the first SCD-related outpatient visit were included as covariates in the relevant multivariable models.

### 2.4. Statistical Analyses

Means (standard deviation; SDs) and frequencies (%) were used to describe all variables. Chi-square tests and *t*-tests were used for categorical and continuous variables, respectively, to assess the differences between the demographic and clinical characteristics of telehealth users and non-users. Multivariable logistic and Poisson regression analyses were conducted to (1) identify patient-level factors associated with all-cause telehealth use and (2) determine if SCD-related telehealth use was associated with the care continuum (≥10 hydroxyurea claims) and healthcare utilization (SCD-related ED visits, hospitalizations), while controlling for covariates (age, sex, regions with an SCD clinic, and prior use of hydroxyurea, ED visits, or hospitalizations). The Box–Tidwell and overdispersion tests were conducted to assess the assumptions of multivariable logistic and Poisson regression analyses, respectively; both assumptions were met (Supplementary Tables S3 and S4). All analyses were conducted using SAS 9.4 (SAS Institute, Cary, NC, USA), with *p* < 0.05 as the significance threshold.

## 3. Results

### 3.1. Patient Characteristics and Differences Between Telehealth Users and Non-Users

A total of 903 children met the inclusion criteria. Table 1 summarizes the key patient characteristics. The overall mean age was 10.4 (±4.1) years in March 2019. Those aged 1–12 years made up the majority (64.1%) of the study subjects. Approximately half of the subjects were males (53.0%), and among those whose race/ethnicity information was available, 79.1% were Black. However, due to the high proportion of missing values (48.1%), the race/ethnicity variable was not included in the multivariable models. About 40% of the patients lived in the five largest cities in Texas (i.e., Houston, Fort Worth, Dallas, Austin, and San Antonio) and 58.2% lived in regions with no pediatric SCD specialist. Seventy percent of the patients had five or more SCD-related visits 1 year prior to the pandemic, including 38.2% with ten or more visits. Of the 903 children, 536 (59.4%) had at least one telehealth visit for any diagnosis between March 2020 and March 2022. No significant difference was found in demographic characteristics between telehealth users and non-users. However, there was a significant difference in the previous SCD-related outpatient visits between the two groups; a larger proportion of telehealth users had 10 or more SCD-related outpatient visits compared to telehealth non-users (*p* = 0.0023).

### 3.2. Patterns of Monthly Outpatient (Telehealth and In-Person) Visits

**Figure 1:**
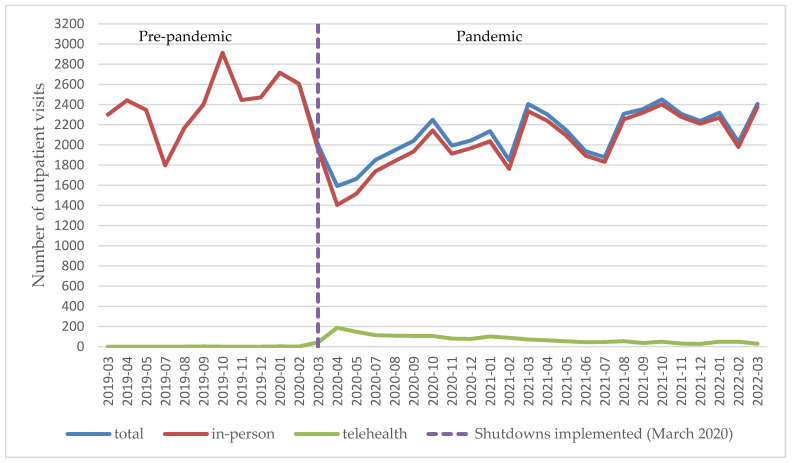
Patterns of monthly number of outpatient visits (in-person, telehealth, and combined) among children with sickle cell disease enrolled in Texas Medicaid from March 2019 to March 2022. June 2020 was excluded due to incomplete data.

Monthly in-person and telehealth visits varied during the study period, as shown in Figure 1. Telehealth use surged between March 2020 and May 2020, then declined but remained above pre-pandemic levels. In the year before the pandemic, there were a total of 26,604 outpatient visits, with only 5 (0.02%) conducted via telehealth. During the first year of the pandemic, among the total of 21,363 outpatient visits, 1153 (5.39%) were telehealth visits. In the second year of the pandemic, telehealth use declined to 571 (2.14%) out of 26,672 outpatient visits. Although telehealth use decreased over time, it remained higher than pre-pandemic levels.

### 3.3. Patient-Level Factors Associated with All-Cause Telehealth Use

Multivariable logistic regression showed that patients with 10 or more pre-pandemic SCD-related outpatient visits had 77.4% higher odds of using telehealth compared to those with 0–4 visits (OR = 1.774, 95% CI = 1.281–2.457, *p* = 0.0006), while controlling for age, sex, and regions with an SCD clinic (Table 2). Other patient-level characteristics, including whether patients lived in regions with an SCD clinic, were not significantly associated with the likelihood of using telehealth.

### 3.4. Association Between SCD-Related Telehealth Use and Care Continuum, and Extensive Healthcare Utilization

Among 536 telehealth users, 442 had one or more SCD-related telehealth visits during the pandemic. Differences between SCD-telehealth users and non-users within this subgroup were similar to those observed for all-cause telehealth users (Supplementary Table S5). Multivariable logistic regression showed that SCD-related telehealth use in the first year of the pandemic was not associated with either having ≥10 hydroxyurea fills during the same period or having an ED visit during one year after the first telehealth visit, after controlling for covariates (Table 3). However, higher odds of ≥10 hydroxyurea fills were associated with pre-pandemic hydroxyurea use (OR: 1.445, 95% CI: 1.377–1.548, *p* < 0.0001) and younger age group (1–12 compared to 13–17 years; OR: 2.563, 95% CI: 1.495–4.394, *p* = 0.0006). ED use within one year after the first SCD-related telehealth visit was significantly associated with ED use 1 year prior to the telehealth visit (OR: 1.921, 95% CI: 1.538–2.399, *p* < 0.0001). Poisson regression results showed that among the 142 patients with one or more ED visits, SCD-related telehealth use was not significantly associated with the number of ED visits during the one year following the first SCD-related telehealth visit. Regression analysis was not performed for hospitalizations due to the very small number of patients with a hospitalization record (fewer than 11), all of whom were non-telehealth users.

### 3.5. Provider Specialty

The major provider specialties associated with all-cause telehealth visits (Table 4) were clinicians in multispecialty clinics (74%), licensed professional counselors (7.1%), and preventive child health specialists (3.9%). For SCD-related telehealth visits (Table 5), the major specialties were clinicians in multispecialty clinics (81%), children’s hospital (5.8%), and clinical nurse specialists (5.3%).

## 4. Discussion

This retrospective cohort study examined the trends, factors, and outcomes associated with telehealth use among children with SCD enrolled in Texas Medicaid before and during the COVID-19 pandemic. Pre-pandemic telehealth use was nearly nonexistent, (0.02%) but surged in the first year of the pandemic before gradually declining in the second year, consistent with findings from previous SCD studies [15,16]. During the pandemic, 59.4% of children with SCD in Texas Medicaid used telehealth, which is a higher rate than in Tennessee (18.4%), Georgia (27.6%), and Michigan (30.4%), but lower than California (82.7%) [16]. It is worth noting that for several patients and providers, the transition to telehealth during the pandemic was more of an obligation than a choice. Thus, while telehealth expanded access to care, the decline in use during the extended pandemic period may reflect the need for in-person assessments, such as physical exams and hemoglobin monitoring, particularly in pediatric patients with SCD. The decline may also reflect patient/caregiver and provider preferences for in-person care over telehealth for disease management. However, due to the lack of information regarding preferences, reasons for visits, or the procedures completed during each visit, this study cannot determine the underlying reason for the decline. Future research is needed to explore these factors and identify strategies for effectively integrating telehealth to improve access to care in this population.

To our knowledge, this is the first study using an adjusted model to identify patient-level factors associated with telehealth use among children with SCD and to examine the geographic proximity to an SCD specialist as a potential predictor. Adjusted analysis showed that having ≥10 SCD-related outpatient visits in the year before the lockdown, a proxy for baseline disease severity, was a key driver of telehealth use after controlling for age, sex, and SCD clinic regions. This finding aligns with the descriptive comparison showing higher pre-COVID outpatient visits among telehealth users with SCD in the four aforementioned states [16]. It suggests that patients with greater disease severity, and thus higher healthcare utilization, were more likely to use telehealth in response to the changes in care delivery brought by the pandemic. This trend was also found in previous studies linking higher pre-COVID healthcare use to greater telehealth engagement [23,24,25]. However, telehealth use did not significantly differ between those living in regions with an SCD specialist and those without, despite a slightly higher proportion of patients living in regions with an SCD specialist using telehealth (43.1% vs. 39.8%, *p* = 0.321). Given that SCD specialists are concentrated in major Texas cities (i.e., Austin, Dallas, Fort Worth, Houston, and San Antonio) and that a higher proportion of telehealth users in California, Michigan, and Tennessee came from areas with high broadband access [16], barriers to telehealth remain for patients most in need.

This is also the first study to examine the association between telehealth use and care continuity in SCD, defined as having ≥10 hydroxyurea fills (indicating 80% adherence) during the first year of the pandemic (March 2020–March 2021). We found no significant association between SCD-related telehealth use and having ≥10 hydroxyurea fills, possibly because people may have been unwilling to pick up prescriptions during the pandemic. However, pre-pandemic hydroxyurea use and younger age (2–12 years) were positively associated with continuity. While no previous studies have examined telehealth’s impact on care continuum in patients with SCD, research on chronic gastrointestinal disease found higher prescription fill rates among telehealth users during the pandemic [26]. This discrepancy could also be explained by differences in disease management. Given the importance of lab monitoring for hydroxyurea, providers may have been cautious about refilling prescriptions without in-person visits, whereas gastrointestinal specialists may have been more comfortable prescribing refills remotely.

We assessed the relationship between ED visits and SCD-related telehealth use using adjusted models (i.e., multivariable logistic and Poisson regression, respectively) and found no association. Similarly to hydroxyurea use, prior ED utilization was a predictor of ED visits during the pandemic, rather than telehealth use itself. This is an important addition to the evidence as the previous study on children with SCD showed higher ED utilization among telehealth users using an unadjusted model [16]. Our finding suggests that the greater utilization of ED was linked to patients’ baseline utilization, which is likely driven by disease severity and access to care, rather than the use of telehealth. A study of commercially insured adults found that follow-up care, including ED visits and hospitalizations, was generally lower after an initial telehealth visit for chronic conditions than after in-person visits [27]. Although prior healthcare utilization was not assessed in the study, patients with initial telehealth encounters tended to have higher diagnostic severity, indicating greater healthcare needs [27]—similar to SCD patients with high pre-pandemic ED use. Due to the low number of hospitalizations in our sample, we did not conduct regression analysis. This may reflect the reduction in hospitalization rates during the pandemic due to stay-at-home policies [28].

Most telehealth visits were provided by clinicians in multi-specialty clinics, but due to limitations in Texas Medicaid provider specialty coding, direct comparisons to other studies—where hematologists [15,16] and behavioral health providers [16] were specifically identified—were not possible. The lack of data on health provider specialties also highlights the need for further research into the availability and accessibility of hematologists by patients with SCD in Texas.

In summary, our findings suggest that telehealth did not independently improve health outcomes among this population. Rather, it was used by patients with a greater need to maintain treatment. The decline in telehealth use after the early months of the pandemic likely reflects both provider and patient preferences for in-person care, given the need for physical exams, screenings, and laboratory monitoring, particularly as SCD symptoms worsen with age. While our results do not establish the value of telehealth as a long-term tool to ensure continuity of care when in-person visits are not feasible, further studies are needed to explore its potential for improving healthcare access, particularly among SCD patients living in rural areas with limited access to specialty care. Efforts to extend the geographic reach of SCD specialists is essential to addressing access barriers. In addition, to support the safer and more effective use of telehealth, future interventions should consider implementing structured triage protocols to guide decisions about when telehealth is clinically appropriate and when in-person visits are warranted. Incorporating patient/caregiver and provider preferences—particularly in light of disease-specific challenges such as stigma and the need for developmental or cognitive monitoring—may further support the integration of telehealth into practical, real-world care delivery.

This study provides valuable insights into patient-level factors and outcomes related to telehealth use, leveraging Texas Medicaid data, which includes the third-largest SCD population in the United States. However, several limitations exist. First, the findings may not generalize to other Medicaid programs, Medicare, commercial insurance, or other healthcare systems. Second, the exclusion of incomplete data for June 2020 may have affected the results. Third, the lack of specificity in provider specialty codes in our database limited our understanding of who provided telehealth services. Fourth, because only ZIP code–level data was available for patients, we were limited in our ability to precisely estimate the distance to the nearest SCD specialty center. As a proxy for proximity to care, we used the “big city” variable. Fifth, low hospitalization rates prevented us from analyzing telehealth’s impact on hospitalizations. Sixth, due to data limitations, we were unable to determine the reasons for the decline in telehealth visits or identify which in-person visits were clinically appropriate for telehealth. We also acknowledge the lack of qualitative data or patient-reported experiences regarding the quality of remote care as a limitation of this study. Lastly, while we adjusted for key confounders, unmeasured factors such as transportation barriers and access to telehealth equipment may still have influenced the results. Future studies using a prospective design and collecting data directly from patients, caregivers, and providers could address these limitations and offer insights into the reasons for telehealth use or non-use in this population. These efforts may also help identify additional barriers to and facilitators of telehealth use in this population.

## 5. Conclusions

Children with SCD often require medical care for pain and complications, making pandemic-related disruptions a potential risk to their health. This study confirmed a rapid increase in telehealth use among this population during the early months of the pandemic and a decline afterwards. Our findings suggest that telehealth did not independently improve health outcomes, but was used by patients with a greater need to maintain treatment. Given that optimal disease management in SCD requires in-person visits, and telehealth can provide a short-term solution when access is limited, extending the geographic reach of SCD specialists is essential to improving access to care.

## Figures and Tables

**Table 1:** Characteristics of children with sickle cell disease enrolled in Texas Medicaid and comparison between all-cause telehealth users and non-users.

Characteristics	Total N = 903 N (%)	Telehealth Users N = 536 N (%)	Telehealth Non-Users N = 367 N (%)	*p*-Value
**Age during study period**				
Mean (SD)	10.4 (4.1)	10.6 (4.1)	10.3 (4.0)	0.334 ^d^
**Age group**				
1–12	579 (64.1)	343 (63.9)	236 (64.3)	0.923 ^e^
13–17	324 (35.9)	193 (36.1)	131 (35.7)	
**Gender**				
Females	424 (47.0)	245 (45.7)	179 (48.8)	0.365 ^e^
Males	479 (53.0)	291 (54.3)	188 (51.2)	
**Race/ethnicity**				
White	19 (2.1)	NR	NR	0.202 ^e^
Black	370 (41.0)	231 (43.1)	139 (37.9)	
Hispanic	79 (8.8)	NR	NR	
Unknown	435 (48.1)	244 (45.5)	191 (52.0)	
**SCD Clinic** (region categories; 10 regions collapsed into ‘Regions with SCD clinic’ vs. other)				
Regions with SCD clinic ^a^	377 (41.8)	231 (43.1)	146 (39.8)	0.321 ^e^
Other	526 (58.2)	305 (56.9)	221 (60.2)	
**Big city** ^b^ (Austin, Dallas, Fort Worth, Houston, San Antonio)				
Yes	355 (39.3)	223 (41.6)	132 (36.0)	0.089 ^e^
No	548 (60.7)	313 (58.4)	235 (64.0)	
**SCD-related outpatient visits 1 year prior to pandemic** ^c^				
0–4 visits	273 (30.2)	141 (26.3)	132 (36.0)	0.0023 ^e^
5–9 visits	285 (31.6)	169 (31.5)	116 (31.6)	
10 or more visits	345 (38.2)	226 (42.2)	119 (32.4)	

Abbreviation: SCD = sickle cell disease; NR = not reported (to meet Center for Medicare and Medicaid Services [CMS] reporting guidelines for suppressing cells <11). ^a^ Houston, Fort Worth, Dallas, Austin, San Antonio, Temple, Galveston, El Paso, Corpus Christi, and Lubbock; information found in the Texas Department of State Health Services website (https://www.dshs.texas.gov/sites/default/files/newborn/pdf/Pediatric-Hematology-Consultants-011724.pdf (accessed on 31 January 2025)). ^b^ Top five metropolitan areas in Texas. ^c^ March 2019–February 2020. ^d^ *p*-value from the Student’s *t*-test. ^e^ *p*-value from the Chi-square test.

**Table 2:** Multivariable logistic regression analysis of the likelihood of having a telehealth visit among children with sickle cell disease enrolled in Texas Medicaid (*n* = 903).

Predictor Variables	Odds Ratio	95% CI		*p*-Value
		Lower	Upper	
Age	1.017	0.984	1.051	0.3224
Female	0.879	0.672	1.149	0.3447
5–9 outpatient visits	1.388	0.992	1.944	0.0560
10+ outpatient visits	1.774	1.281	2.457	0.0006
Region with SCD clinic	1.130	0.860	1.484	0.6318

Abbreviation: CI = Confidence Interval. Reference categories: male; 0–4 outpatient visits; and living in a region without an SCD clinic.

**Table 3:** Multivariable regression analyses of hydroxyurea and healthcare resource utilization during the pandemic among children with sickle cell disease enrolled in Texas Medicaid.

	10 or More Hydroxyurea Fills (N = 900)				Emergency Department (ED) Visit (N = 903)				Number of ED Visits (N = 142)			
	OR	95% CI		*p*-Value	OR	95% CI		*p*-Value	RR	95% CI		*p*-Value
		Lower	Upper			Lower	Upper			Lower	Upper	
SCD-related telehealth visit(s) during 1-year post-pandemic	1.143	0.700	1.865	0.5936	1.254	0.866	1.817	0.2309	1.148	0.862	1.530	0.3455
1–12 years old	2.563	1.495	4.394	**0.0006**	0.888	0.607	1.299	0.5416	0.884	0.664	1.178	0.4004
Female	0.954	0.584	1.558	0.8507	0.923	0.638	1.336	0.6703	1.063	0.794	1.424	0.6802
Region with SCD clinic	1.358	0.833	2.216	0.2199	0.744	0.508	1.090	0.1286	1.187	0.891	1.582	0.2409
Pre-pandemic utilization (SCD-related)												
Hydroxyurea use	1.445	1.377	1.548	**<0.0001**	-	-	-	-	-	-	-	-
Evidence of prior ED visit	-	-	-	-	1.921	1.538	2.399	**<0.0001**	-	-	-	-
Number of prior ED visits	-	-	-	-	-	-	-	-	1.120	0.994	1.262	0.0625

Abbreviation: CI = confidence interval; OR = odds ratio from logistic regression analyses; RR = relative risk from Poisson regression analysis; SCD = sickle cell disease. Reference categories: No SCD-related telehealth visit 1 year post-pandemic; 13–17-year-olds; male; living in a region without an SCD clinic; and no evidence of prior ED visit. Bolding indicates statistical significance.

**Table 4:** Provider specialties for all-cause telehealth visits.

Provider Specialty	Frequency (n)	Percent (%)
Multispecialty Clinic	1675	73.82
Licensed Professional Counselor	160	7.05
Early and Periodic Screening, Diagnosis, and Treatment	89	3.92
Clinical Nurse Specialist	68	3.00
Children’s Hospital	64	2.82
Pediatrics	54	2.38
Mental Health (MH) Case Mgt/MH Rehab/YES waiver	46	2.03
Speech Therapy (CCP)	14	0.62
MH Case Management/MH Rehabilitative Services—Non-LMHA	13	0.57
Dentists/Orthodontists	12	0.53
Licensed Clinical Social Worker	12	0.53
Psychologist	<11	NR
Teaching Affiliate	<11	NR
Home Health Agency	<11	NR
Physician Medicine and Rehabilitation	<11	NR
State Psychiatric Hospital	<11	NR
Psychiatry	<11	NR
CCP Provider (WIC—Immunizations Only, Milk Donor)	<11	NR
Ambulatory Surgical Services	<11	NR
Genetics	<11	NR
Rural Health Clinic (Independent)	<11	NR
Physician Assistant	<11	NR
Internal Medicine	<11	NR
Neurology	<11	NR
Hospital—Profit/Acute/101 Plus Beds	<11	NR

Abbreviations: CCP= Crisis Counseling Assistance and Training Program; WIC = women, infants, and children; YES = youth empowerment services; NR = not reported.

**Table 5:** Provider specialties for SCD-related telehealth visits.

Provider Specialty	Frequency (n)	Percent (%)
Multispecialty Clinic	765	81.12
Children’s Hospital	55	5.83
Clinical Nurse Specialist	50	5.30
Early and Periodic Screening, Diagnosis, and Treatment	30	3.18
Pediatrics	14	1.48
Teaching Affiliate	<11	NR
State Psychiatric Hospital/Long-Term or Specialized Care/Rehabilitation Hospital	<11	NR
Dentists/Orthodontists	<11	NR
Home Health Agency	<11	NR
Physician Assistant	<11	NR
Internal Medicine	<11	NR
Neurology	<11	NR
Genetics	<11	NR
Rural Health Clinic (Independent)	<11	NR

Abbreviations: NR = not reported (to meet Center for Medicare and Medicaid Services [CMS] reporting guidelines of suppressing cells < 11).

## Data Availability

Restrictions apply to the datasets: the datasets presented in this article are not readily available due to privacy protections under the Data Use Agreement between Texas Health and Human Services and The University of Texas at Austin College of Pharmacy Health Outcomes Division.

## Supplementary Materials

The following supporting information can be downloaded at: https://www.mdpi.com/article/10.3390/healthcare13131519/s1, Table S1: Patient attrition; Table S2: Teleservices procedure codes; Table S3: Box-Tidwell test for age as a continuous variable in logistic regression analysis. Table S4: Testing for overdispersion in Poisson regression analysis. Table S5: Characteristics of children with sickle cell disease enrolled in Texas Medicaid and comparison between SCD-related telehealth users and non-users.
