# Supplementary material for: Telehealth Use Among Medicaid-Enrolled Children with Sickle Cell Disease Before and During the COVID-19 Pandemic
Source: Healthcare (Basel). 2025 Jun 25;13(13):1519. doi: 10.3390/healthcare13131519 (PMC12249286; doi:10.3390/healthcare13131519)
Supplement: Supplementary file 1 [file healthcare-13-01519-s001.zip › healthcare-3618657-supplementary.pdf]

SUPPLEMENTARY MATERIALs

Table S1. Patient attrition

**Inclusion Criteria**	**Number of patients included**
Patients with at least one sickle cell disease diagnosis	10,075
Individuals with at least 3 hospitalizations or outpatient visits	5,393
No cancer diagnosis at any point in study period	5,055
Individuals aged between 1 and 18 years during the study period (03/01/19-03/31/22)	1,863
No dual eligibility for Medicaid and Medicare	1,863
Individuals continuously enrolled for 3 years and 1 month (i.e., 03/2019-02/2020; 03/2020-02/2021; 03/2021-03/2022)	903
**Final sample**	**903**

Table S2. Teleservices Procedure Codes

**Definition**	**Codes**
"Teleservices" includes audio/visual services done by physicians (telemedicine) or non-physicians (telehealth), as well as audio-only done by any licensed provider.	Teleservices before 9/1/2019 include procedure codes G0406, G0407, G0408, G0425, G0426, G0427, G0459, or 99457 regardless of modifier or procedure codes 90791, 90792, 90801, 90802, 90804, 90805, 90806, 90807, 90808, 90809, 90832, 90833, 90834, 90836, 90837, 90838, 90862, 90951, 90952, 90954, 90955, 90957, 90958, 90960, 90961, 92507, 92508, 92521, 92522, 92523, 92524, 97150, 97165, 97166, 97167, 97168, 97530, 97802, 97803, 97804, 99201, 99202, 99203, 99204, 99205, 99211, 99212, 99213, 99214, 99215, 99241, 99242, 99243, 99244, 99245, 99251, 99252, 99253, 99254, 99255, 99354, 99355, 99356, 99357, G0406, G0407, G0408, G0425, G0426, G0427, G0459, M0064, S9152, or S9470 with modifier 'GT' or '95'.<br><br>Beginning 9/1/2019, teleservices include any procedure codes with modifier GT or 95 or procedure codes G0406, G0407, G0408, G0425, G0426, G0427, G0459, 99457, 99441, 99442, or 99443 regardless of modifier.

Table S3. Box-Tidwell Test for age as a continuous variable in logistic regression analyses

Analysis of Maximum Likelihood Estimates					
**Parameter**	**DF**	**Estimate**	**Standard Error**	**Wald Chi-Square**	**Pr > ChiSq**
**Intercept**	1	0.7418	0.8445	0.7715	0.3797
**age**	1	-0.1763	0.2990	0.3477	0.5554
**age*log_age**	1	0.0584	0.0906	0.4154	**0.5192**

Interaction term p-value >0.05, thus linearity assumption was met.

Table S4. Testing for overdispersion in Poisson regression analysis

Criteria For Assessing Goodness Of Fit			
**Criterion**	**DF**	**Value**	**Value/DF**
**Deviance**	136	40.0380	**0.2944**
**Scaled Deviance**	136	40.0380	0.2944
**Pearson Chi-Square**	136	49.6200	**0.3649**
**Scaled Pearson X2**	136	49.6200	0.3649
**Log Likelihood**		-129.0417	
**Full Log Likelihood**		-175.6139	
**AIC (smaller is better)**		363.2277	
**AICC (smaller is better)**		363.8499	
**BIC (smaller is better)**		380.9627	

Deviance and Pearson Chi-Square estimates (value/DF) are substantially lower than 1, thus Poisson model does not indicate overdispersion.

Table S5. Characteristics of children with sickle cell disease enrolled in Texas Medicaid and comparison between SCD-related telehealth users and non-users

Characteristics	Total N=903 N (%)	SCD-related telehealth users N=442 N (%)	SCD-related telehealth non-users N=461 N (%)	P-value
**Age during study period**				
Mean (SD)	10.4 (4.1)	10.6 (4.1)	10.3 (4.1)	0.249[d]
**Age group**				
1-12	579 (64.1)	282 (63.8)	297 (64.4)	0.845[e]
13-17	325 (35.9)	160 (36.2)	164 (35.6)	
**Sex**				
Female	424 (47.4)	208 (47.1)	216 (46.8)	0.951[e]
Male	479 (52.6)	234 (52.9)	245 (53.2)	
**Race/ethnicity**				
White	19 (2.1)	NR	11 (2.4)	0.421[e]
Black	370 (41.0)	193 (43.7)	177 (38.4)	
Hispanic	79 (8.8)	38 (8.6)	41 (8.9)	
Unknown	435 (48.1)	203 (45.9)	232 (50.3)	
**SCD Clinic** (10 regions collapsed into 'Regions with SCD clinic' vs. Other)				
Region with SCD clinic[a]	377 (41.8)	182 (41.2)	195 (42.3)	0.732 [e]
Other	526 (58.2)	260 (58.8)	266 (57.7)	
**Big city**[b] (Houston, Fort Worth, Dallas, Austin, San Antonio)				
Yes	355 (39.3)	175 (39.6)	180 (39.1)	0.866 [e]
No	548 (60.7)	267 (60.4)	281 (60.9)	
**SCD-related outpatient visits 1-year prior to pandemic**[c]				
0-4 visits	273 (30.2)	95 (21.5)	178 (38.6)	<0.0001 [e]
5-9 visits	285 (31.6)	151 (34.2)	134 (29.1)	
10 or more visits	345 (38.2)	196 (44.3)	149 (32.3)	

Abbreviation: SCD = sickle cell disease

[a] Houston, Fort Worth, Dallas, Austin, San Antonio, Temple, Galveston, El Paso, Corpus Christi, and Lubbock; information found in the Texas Department of State Health Services website (https://www.dshs.texas.gov/sites/default/files/newborn/pdf/Pediatric%20Hematology%20Consultants011724.pdf) as of 1/31/2025

[b] Top 5 metropolitan area in Texas

[c] March 2019 - February 2020

[d] p-value from the Student's t-test

[e] p-value from the Chi-square test
